# Ocular chemical burn associated with gel type alcohol-based hand sanitizer: A case report

**DOI:** 10.1097/MD.0000000000027292

**Published:** 2021-10-22

**Authors:** Jaekyoung Lee, Jong Hwa Jun

**Affiliations:** Department of Ophthalmology, Keimyung University Dongsan Medical Center, Daegu, Republic of Korea.

**Keywords:** alcohol-based hand sanitizer, burn, chemical, ocular surface

## Abstract

**Introduction:**

Alcohol-based hand sanitizers (ABHS) are widely used for hand hygiene due to the coronavirus disease pandemic. However, risk awareness regarding its adverse effects is lacking. We aim to report a case of ocular chemical burn that showed severe clinical presentation associated with ABHS.

**Patient concerns:**

A 5-year-old girl presented with severe left eye pain after 62% gel-type ABHS splashed into her eye.

**Diagnosis:**

On slit lamp examination, a near total corneal and conjunctival epithelial defect with limbal pale on the lower half of the cornea was noted. Severe ocular burn by ABHS was prominent with suspected limbal stem cell damage.

**Interventions:**

She was hospitalized and was prescribed topical medications including antibiotics, steroid eye drops with preservative-free artificial tears, and oral nonsteroidal anti-inflammatory drugs.

**Outcomes:**

Despite intensive medical treatments, the corneal and conjunctival epithelial defects showed no improvement up to the 4^th^ hospital day. After additional instillation of autoserum eye drops to promote epithelial healing, the corneal epithelium barely recovered from the temporal limbus. On the third week of admission, the epithelial defect was completely resolved without corneal opacity, although with minimal symblepharon in the lower fornix.

**Conclusions:**

Gel-type ABHS can cause severe form of ocular chemical burn such as delayed ocular surface healing. In clinical setting, immediate and thorough rinsing of alcohol-based gel and early intensive treatment are crucial.

## 1 Introduction

Since the world is facing the coronavirus disease pandemic, the use of hand sanitizers has become routine to prevent the spread of infection.^[1]^ The Center for Disease Control recommends washing hands with soap for 20 seconds for hand hygiene and prevention of infection; however, alcohol-based hand sanitizers (ABHSs) with 60% to 90% alcohol may be used alternatively if soap and water are unavailable.^[2,3]^ Although not as effective as washing with soap, the ABHSs have gained popularity due to their accessibility to the general public.^[1]^ However, despite their popularity, there is lack of awareness regarding the risks of ABHSs.

If overused, the ABHSs can result in toxic, allergic effects and irritation on the skin and eyes.^[4–6]^ The aerosolized alcohols and constituent chemicals can serve as irritants, causing allergic contact dermatitis, ocular surface discomfort, and precorneal tear film alterations.^[4–6]^ Direct contact with ocular tissues can result in chemical burns similar to those from conventional alcohol solutions.^[7,8]^ Previous studies have reported that exposure to high concentration of ethanol (over 50%) can cause loss of corneal epithelial cells and stromal keratocytes with corneal inflammation and edema, leading to severe ocular burns.^[9–11]^

Ocular chemical burn is a true ocular emergency, causing significant damage to the ocular surface and may result in permanent visual morbidity and sequelae.^[7,8]^ However, most alcohol burn cases occur after exposure to low concentration or liquid type of alcohols, which rarely leave visual morbidities or treatment delay.^[12–14]^ We encountered a case of a 5-year-old child with a severe clinical presentation of an ocular burn resulting from gel-type ABHS. Therefore, we aim to discuss the risk of gel-type ABHS, and inform clinicians that gel-type ABHS can induce ocular chemical burns.

## 2 Case presentation

**Figure 1:**
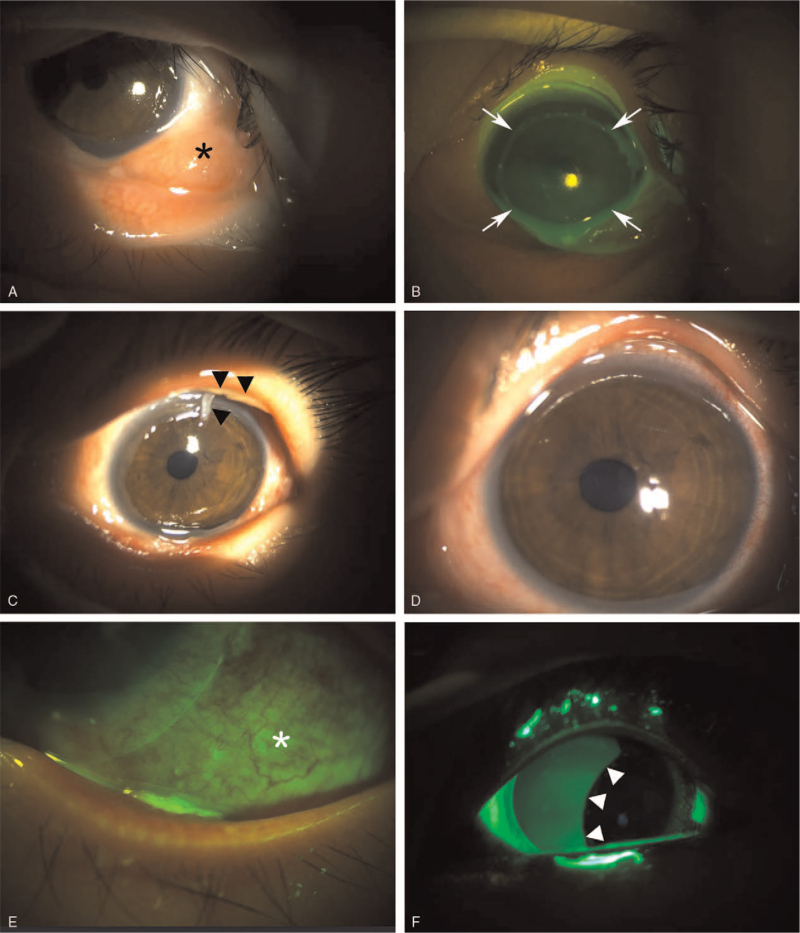
(A) Slit lamp photograph upon initial presentation. Severe hyperemia, chemosis (asterisk), and limbal pale on the lower half of the cornea were noted. (B) Fluorescein staining revealed a near total corneal epithelial defect (arrows). (C) Two d after admission, necrotic debris started to form a pseudomembrane at the palpebral conjunctiva (black arrow heads). (D) Despite instillation of autoserum eye drop to promote epithelial healing, the corneal epithelial defect showed no improvement at the 6^th^ day of admission. (E) Fluorescein staining revealed the remaining widespread conjunctival epithelial defect (white asterisk) at the 6^th^ day of admission. (F) Corneal epithelium started to recover from temporal limbus but a widespread epithelial defect of the nasal corneal epithelium was still noted (white arrow heads) at the 8^th^ day of admission.

A 5-year-old girl visited our clinic for severe left eye pain. Four hours prior to arrival, she reached out to use a gel-type ABHS of 62% concentration, which was placed at average adult height in an elevator. As soon as she tried to reach for the sanitizer, the alcohol gel splashed into her left eye.^[15]^ Her parents initially rinsed her eye with tap water. However, 2 hours later, she complained of severe left eye pain, photophobia, tearing, and eyelid swelling, which prompted a visit to our clinic. The best-corrected visual acuity of her left eye was 20/100, and the intraocular pressure was 20 mm Hg. After immediate copious irrigation with 2 L of 0.9% normal saline, slit-lamp examination revealed eyelid swelling, severe chemosis, moderate conjunctival injection with limbal pale on the lower half of the cornea from the 3 to 9 o’clock position (Fig. 1A). There was also an extensive epithelial defect of the cornea and conjunctiva (Fig. 1B). Although close daily follow-up under admission was recommended, her parents refused admission. She was followed up in our outpatient clinic.

On the next day, since there was no improvement of the epithelial defects in the cornea and conjunctiva, and limbal pale persisted, she was admitted to our hospital. Topical medication were prescribed with oral nonsteroidal anti-inflammatory drugs as follows; levofloxacin 0.5% (Cravit ophthalmic solution, Santen, Osaka, Japan) bihourly, prednisolone acetate 1.0% (Pred-forte, Allergan, Rochester, NY) bihourly, preservative-free artificial tears 6 times daily, and solcoseryl concentrate (Solcorin ophthalmic gel, Hanlim Pharm. Co., Seoul, Korea) at every bed time. The corneal epithelial defect did not improve for 2 days after admission, and the necrotic debris of the palpebral conjunctiva formed a pseudomembrane (Fig. 1C). Despite the addition of autoserum eye drop 6 times a day, there was no sign of corneal, conjunctival epithelial defect recovery. Furthermore, necrotic epithelial deposits were observed (Fig. 1D and 1E) on the 6^th^ hospital day. To promote epithelial healing, 1% prednisolone acetate was tapered to 4 times a day.

**Figure 2:**
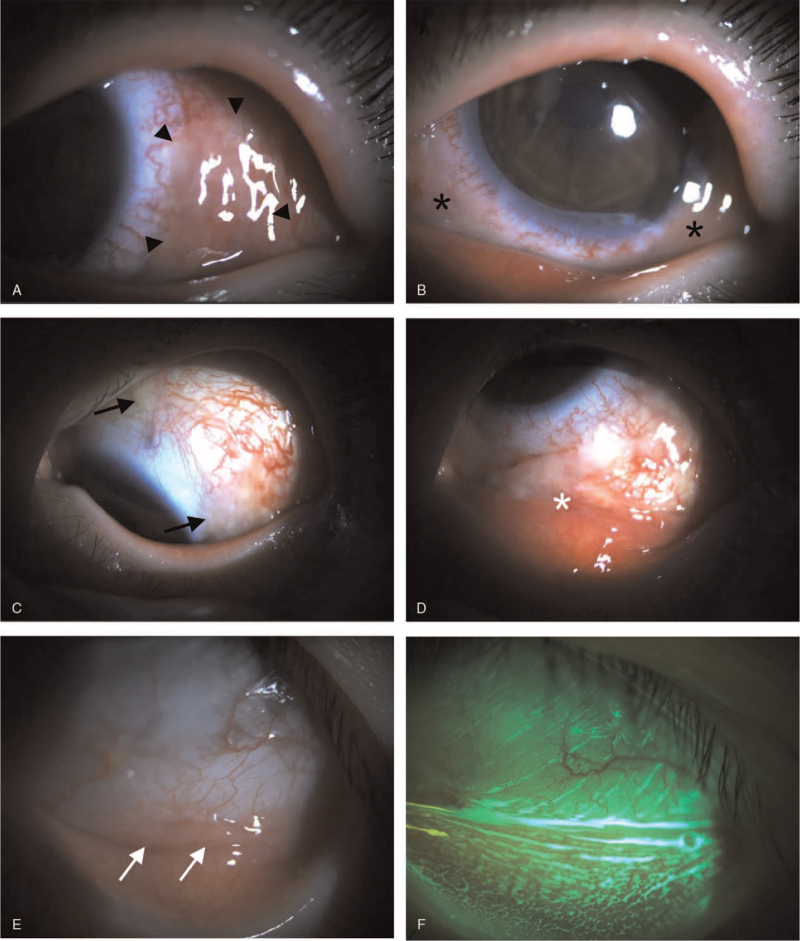
(A) and (B) On the 8^th^ day of admission, while corneal epithelial defect started to improve, severe injection and edema of the conjunctiva were noted with necrotic tissues (black arrow heads and asterisks). (C) and (D) Conjunctival tissues showed focal adhesions (black arrows) and symblepharon (white asterisks) at the 15^th^ day of admission. (E) and (F) Three wk after admission, the corneal and conjunctival epithelial defect recovered completely with minimal inferior fornix symblepharon (white arrows).

On the 8^th^ day of admission, corneal epithelial healing was seen in the temporal limbus (Fig. 1F); however, severe inflammation of the conjunctiva with necrotic debris and pseudomembrane was observed (Fig. 2A and 2B). One week later, despite the continuous eye drop regimen, and recovering corneal epithelium, we noted adhesion of the conjunctival tissues and symblepharon at the inferior fornix (Fig. 2C and 2D). Three weeks later, her best-corrected visual acuity improved to 20/30 and the corneal epithelium was completely healed with no corneal opacity; but with focal symblepharon at the lower fornix (Fig. 2E and 2F). The endothelial cell density was 2841 cells/mm^2^, and showed no abnormality.

## 3 Discussion and conclusions

Alcohol is widely used in ophthalmic surgeries such as photorefractive keratectomy, treatment for recurrent corneal erosion syndrome, and excision of pterygium.^[8,16–18]^ In most cases, application of 20% ethanol for 30 to 40 seconds to the corneal surface can damage the corneal epithelium and result in epithelial debridement.^[16–18]^ However, it is well known that high concentration of ethanol can cause deep coagulation to the corneal epithelium or stromal keratocyte, with severe corneal inflammation.^[9–11]^ This case involved an accidental exposure to gel type ABHS.

The chemical agent in our case, which led to severe burning, was 62% ethanol in gel form. Our case report has 2 valuable implications for ophthalmologists: the causative agent was a gel-type ethanol, which caused a severe clinical course as compared to conventional alcohol chemical burn; and this case demonstrated delayed epithelial healing of the cornea and conjunctiva.

Previously reported cases of ocular burn after alcohol exposure primarily involved either liquid or aerosol alcohol.^[12–14,19]^ The exposure of cornea with liquid or aerosol type of alcohol is relatively short due to instant blinking and washing out or dilution with tears as soon as it touches the cornea. Therefore, these types of alcohols leave only mild ocular irritation and superficial epithelial defects. However, the gel-type ABHS has a higher viscosity, hence may cause longer exposure to ocular tissues with delayed washout. The same principle is seen in an ointment with a longer contact time than eye drops.^[20]^ In our case, this resulted in a deeper and wider range of initial damage to the epithelium and limbus, and induced deep penetration, leading to slow corneal and conjunctival epithelial defect recovery.

Due to the rapid recovery seen in previous reports with a corneal epithelial defect after following alcohol exposure,^[12–14]^ we expected a quick epithelial healing and less conjunctival inflammation in our patient. However, in contrast to our expectations, there was no sign of corneal and conjunctival recovery for 7 hospital days.

Several studies reported the effects of alcohol exposure to ocular tissues. Oh et al^[9]^ reported that ethanol decreases the viability of cells in a concentration-dependent manner by causing cell lysis, suppression of proliferation, and increase in the expression of pro-inflammatory cytokines in the epithelial and stromal cells. There have been reports that exposure to 50% ethanol may cause keratocyte loss and corneal edema,^[10]^ and 100% ethanol results in great decrease in keratocyte count with acute corneal inflammation.^[11]^ Similarly, in our case, the 62% gel type ethanol caused extensive defect on the corneal and conjunctival epithelial cells. In addition, it caused dysfunction and damage to the limbal stem cells. In fact, approximately a week after treatment initiation, limbal stem cell deficiency was suspected due to prolonged epithelial defect. Fortunately, a few days later with addition of autoserum eye drop, the corneal and conjunctival epithelium started to recover. Although the superficial limbal stem cells had been damaged, the very deep-seated limbal stem cells were likely to have grown to the surface or some of the remaining stunned stem cells recovered.

Peng et al^[19]^ reported a case report of deep corneal endothelial injury following an exposure to alcohol anti-mist agent. At 1-year follow-up, the patient showed stromal opacity and loss of endothelial cell density to 1500 cells/mm^2^. Comparatively, in our case, an assumption may be made that the penetration of the ABHS was not that deep. However, ocular burn after gel-type ABHS can show delayed epithelial healing process, thus clinical attention is warranted. Moreover, due to the slow recovery of the corneal epithelium, amniotic membrane transplantation may be considered as an early treatment in patients except for children at risk of amblyopia.^[21,22]^ Since our patient was a 5-year-old child, we considered amniotic membrane transplantation at 1 week following the trauma. However, it was not carried out owing to the possibility of amblyopia by long retention of amniotic membrane.

In conclusion, the increased public use and popularity of gel-type ABHSs can be threatening especially in cases where the ABHS is homemade based on unreliable texts or videos from the internet. When ABHSs are exposed to mucosal surfaces, especially the eye, the result can be disastrous. Here, we present a unique case of chemical ocular burn after an exposure to 62% ethanol-based gel type hand sanitizer to raise the awareness of the potential hazards of AHBS, and demonstrate that this type of chemical burn has a delayed course of corneal and conjunctival epithelium healing.

## Author contributions

**Investigation:** Jaekyoung Lee.

**Supervision:** Jong Hwa Jun.

**Writing – original draft:** Jaekyoung Lee.

**Writing – review & editing:** Jong Hwa Jun.
